# Alkali-Activated Polymers for Grouting: A Review of Mechanisms, Performance, and Engineering Applications

**DOI:** 10.3390/polym18050650

**Published:** 2026-03-06

**Authors:** Beining Liu, Mengtang Xu

**Affiliations:** 1 Yangzhen Area Office of Shunyi District, Beijing 101309, China; 2 School of Civil Engineering, Beijing Jiaotong University, Beijing 100044, China; 3 School of Mining Engineering, Guizhou Institute of Technology, Guiyang 550003, China

**Keywords:** alkali-activated polymers, grouting, reaction mechanism, mechanical property

## Abstract

Under dual challenges of global infrastructure expansion and industrial solid waste management, alkali-activated polymers (AAP), as industrial solid-waste-based low-carbon cementitious materials, exhibit immense potential in grouting engineering applications. This review synthesizes current research progress through three critical dimensions: reaction mechanisms, performance characteristics, and grouting applications (grouting for reinforcement and water-blocking). The reaction mechanism universally comprises three stages: dissolution, depolymerization, and polycondensation. Key performance determinants include precursor composition (e.g., slag, fly ash, metakaolin) and alkaline activator properties (type, modulus, concentration). The multifunctional advantages of AAP are fundamentally governed by their microstructural evolution. Specifically, the rapid formation of highly cross-linked C-(A)-S-H and N-A-S-H gels directly contributes to rapid setting and high early strength development, with high-calcium precursors such as slag exhibiting faster strength gain than low-calcium systems, such as fly ash and metakaolin. Furthermore, the absence of vulnerable calcium hydroxide phases, combined with a densified, low-porosity aluminosilicate network, provides superior thermal stability, corrosion resistance, frost durability, and low permeability. Nevertheless, pronounced autogenous shrinkage and drying shrinkage, driven by mesopore moisture loss and the highly viscoelastic solid skeleton, remain primary constraints for field implementation. In grouting reinforcement, AAP can effectively enhance the strength and structural integrity of weak soils, such as soft clay, loess, and sulfate-rich saline soils. For grouting water-blocking, particularly in sodium-silicate-based binary systems, AAP achieves rapid gelation, superior washout resistance, and high anti-seepage pressure, proving optimal for groundwater inflow control. Future research must prioritize (i) standardized mix design protocols for performance consistency, (ii) advanced shrinkage mitigation strategies, (iii) systematic durability assessment under coupled environmental stressors (e.g., wet–dry cycling, chemical attack, thermal fatigue), and (iv) cross-disciplinary collaboration for industrial-scale validation.

## 1. Introduction

**Figure 1:**
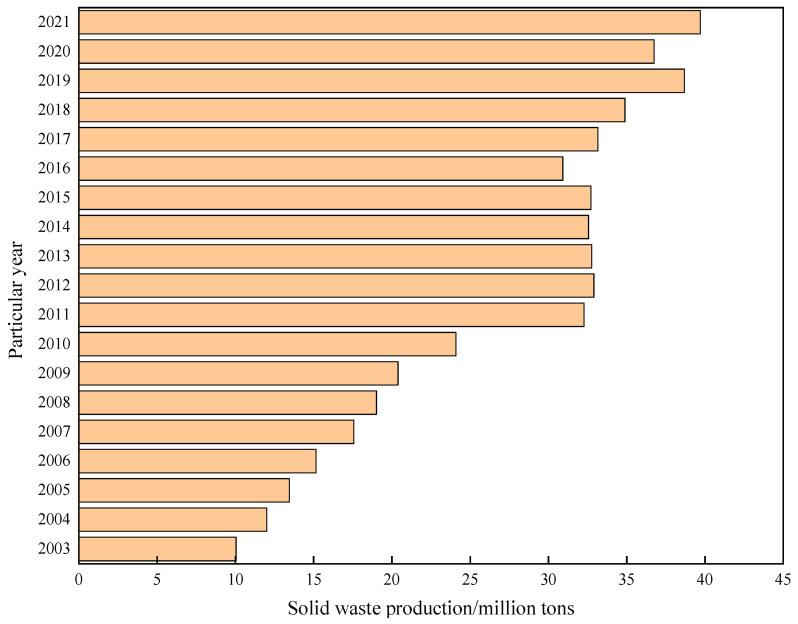
Annual output of solid waste in China. Reprinted with permission from ref. [11].

Since the invention of Portland cement by Joseph Aspdin in 1824, silicate cement and its concrete have been widely applied in civil engineering, highways, railways, water conservancy, marine engineering, and aerospace, becoming the most extensively used and highest-volume construction material globally [1]. Due to increasing global infrastructure investment, underground construction projects have surged. During such construction, weak soil strata or fractured rock layers are frequently encountered, necessitating large quantities of grouting materials to reinforce and improve weak soils and control groundwater seepage issues. Traditional cement materials require substantial consumption of resources and energy during production and emit large amounts of harmful gases, such as CO_2_, CO, NO_x_, and SO_2_. These limitations, including high energy consumption and high pollution emissions, inevitably pose serious threats to the ecological environment [2,3]. Concurrently, the production process generates vast quantities of industrial solid wastes, including slag, fly ash, steel slag, red mud, etc. [4,5,6,7]. By the end of November 2024, its annual production exceeded 3.85 billion tons [3,8,9,10]. Taking China as an example, Figure 1 shows the annual production statistics of solid waste in China. Stockpiling these industrial solid wastes not only wastes land resources but also poses pollution risks to soil and groundwater. Therefore, the research and development of eco-friendly construction cementitious materials characterized by low energy consumption, low pollution, high stability, and high durability are urgently needed.

**Figure 2:**
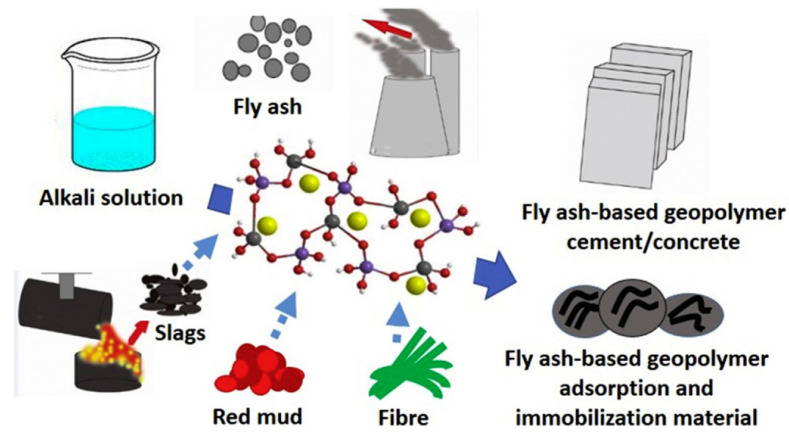
The production process of fly ash-based polymers. The blue arrows indicate the material flow and reaction direction. In the central molecular structure, the colored spheres represent different elements (e.g., Si, Al, O, Na). Reprinted with permission from ref. [17].

In 1940, Belgian scientist Purdon first produced a novel fast-hardening cementitious material by adding alkali to slag [12]. In the 1950s, the Soviet Union developed a new type of alkali-activated slag cement, subsequently conducting extensive production trials and achieving industrial-scale production. Compared to Portland cement, alkali-activated slag cement offers advantages such as low water demand, low heat of hydration, high strength, and good durability. However, it also suffers from fatal shortcomings like rapid setting and hardening and high drying shrinkage of the hardened paste [13,14]. With further research into materials, cementitious materials formed by activating precursors—such as industrial by-products (slag, fly ash, steel slag, etc.) and natural minerals (metakaolin, volcanic ash, calcined clay, etc.)—with alkaline activators (e.g., sodium hydroxide, sodium silicate, sodium carbonate, etc.) have gradually gained wider application. This category of inorganic materials, characterized by a polymeric structure, is known as alkali-activated polymers. Due to their unique three-dimensional spatial network structure, alkali-activated polymers exhibit many superior properties difficult to achieve with pure cement-based materials particularly outstanding in mechanical performance, heat resistance, corrosion resistance, freeze–thaw resistance, impermeability, and adjustable setting time. Alkali-activated polymers not only fully utilize industrial solid wastes as raw materials, enabling large-scale resource recycling of these wastes, but also feature a simple production process and very low energy consumption during manufacturing, reducing CO_2_ emissions by 60% to 80%. Given that the construction sector accounts for approximately 8% of global CO_2_ emissions, the large-scale adoption of AAP in infrastructure projects—particularly in grouting engineering involving massive material consumption—represents a meaningful pathway toward carbon neutrality. Furthermore, the simultaneous valorization of industrial solid wastes addresses both environmental pollution and resource scarcity, delivering dual ecological and economic benefits. They represent a class of advanced green building materials with immense development potential [15,16,17]. Recently, the application of environmentally friendly geopolymers and related advanced polymer composites has successfully expanded into diverse structural domains, ranging from underground infrastructure systems such as fiber-reinforced geopolymer concrete pipes [18] to advanced structural components such as pultruded fiber-reinforced polymer beams [19], demonstrating excellent structural integrity and durability. For example, the production process of fly-ash-based alkali-activated polymers is shown in Figure 2 [17].

In recent years, significant achievements have been made in the theoretical research on alkali-activated polymer cementitious materials and their application in the construction engineering field. However, existing reviews have predominantly focused on structural applications, such as concrete and surface coatings, leaving a critical gap in systematic coverage of AAP for grouting engineering. Grouting presents unique technical demands—including rapid gelation, high injectability, washout resistance under dynamic groundwater conditions, and long-term durability in confined subsurface environments—that differ fundamentally from conventional construction scenarios. The absence of a dedicated review addressing these grouting-specific requirements hinders the rational design and field implementation of AAP-based grouts. To bridge this gap, this paper presents a comprehensive review of AAP for grouting applications, organized around three interconnected dimensions: reaction mechanisms, fundamental performance characteristics, and grouting applications including reinforcement and water-blocking. To promote the application of alkali-activated polymer materials in grouting engineering, this paper reviews and summarizes research progress in alkali activation reaction mechanisms, fundamental properties, and grouting applications (including reinforcement and water-blocking). Firstly, the raw materials, alkali activators, and reaction mechanisms of alkali-activated polymers are introduced, which can be fundamentally divided into three stages: dissolution, depolymerization, and polycondensation. Secondly, the properties of alkali-activated polymers (mechanical properties, workability, and setting time) and the key influencing factors are systematically reviewed. These factors include the types, content, and concentration of raw materials and alkali activators, water content, and curing conditions. Finally, recent research progress and existing problems in the application of alkali-activated polymers in grouting in recent years are discussed, and future research directions with application prospects are proposed.

## 2. Reaction Mechanisms

Researchers worldwide have conducted a series of studies on alkali-activated materials. In the 1940s, Purdon proposed the earliest alkali activation reaction mechanism. He suggested that NaOH acts as a catalyst during cement hardening: NaOH dissolves the silico-aluminates in the cement, forming sodium silicate and sodium meta-aluminate. These then further react with Ca(OH)_2_ to generate calcium silicate and calcium aluminate gels while regenerating NaOH to catalyze the next reaction cycle [12]. Possessing excellent properties such as rapid hardening and high strength [20], high-temperature resistance [21], and acid corrosion resistance [22,23], this novel cementitious material free from Portland cement clinker has become a global research hotspot. With the deepening of research, precursor systems have expanded from natural minerals like kaolin [24,25,26,27] to various industrial and municipal solid wastes, including slag [28,29], furnace slag [30], steel slag [31], fly ash [32,33,34,35], and municipal sludge [36]. Concurrently, alkaline activators have evolved from single-component types like hydroxides [24], sodium silicate (water glass) [37,38], and carbonates [29] to multi-component composite activators [39,40,41]. The enrichment of raw material sources and broadening of selection options have significantly propelled application development. Corresponding preparation techniques have progressively matured for different alkali-activated systems. Currently, significant progress has been made in research on key parameters such as precursor type, activator type, dosage, and water content influencing material properties.

The raw materials for alkali-activated cementitious materials can be classified into high-calcium silico-aluminate materials (such as slag) and low-calcium silico-aluminate materials (such as fly ash, kaolin, etc.) based on their calcium content. The calcium content directly influences the strength and reaction kinetics of the alkali-activated polymers. For different raw materials, the optimal activator type and dosage vary significantly. For instance, some scholars [42] have proposed that for alkali-activated slag cementitious materials, sodium silicate (water glass) is more effective than NaOH in activating slag. The dosage of the alkaline activator plays a decisive role in the strength of alkali-activated slag cementitious materials. Research [43] indicates that strength initially increases and then decreases with increasing alkali dosage, suggesting an optimal range exists. For metakaolin and fly ash, studies by Palomo et al. [40] and Duxson et al. [41] found that a composite activator of NaOH and sodium silicate yields the best activation effect. Davidovits [44] discovered that for a composite NaOH/sodium silicate activator, a silica modulus (SiO_2_/Na_2_O ratio) of the sodium silicate component of 1.85 resulted in higher strength and better durability for alkali-activated metakaolin materials. Beyond strength, workability properties like fluidity [45] and setting time [46] serve as key metrics for mix proportion optimization. Synthesizing these findings reveals substantial variations in critical parameters (e.g., silica modulus, alkali dosage) across proposed optimal mixes, even for identical material types. This inconsistency stems from source-dependent differences in chemical composition, hindering unified consensus.

**Figure 3:**
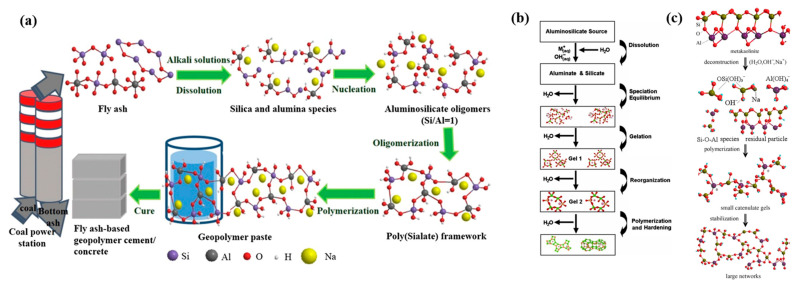
Conceptual models of the geopolymerization process. (**a**) Specific reaction pathway for fly-ash-based geopolymers (reprinted with permission from ref. [17]); (**b**) general theoretical framework (reprinted with permission from ref. [48]); (**c**) specific reaction pathway for metakaolin-based geopolymers (reprinted with permission from ref. [49]).

The analysis of alkali activation reaction mechanisms is integral to understanding the preparation and fundamental properties of alkali-activated cementitious materials. Consequently, diverse models have been proposed to describe these processes. Glukhovsky delineated a linear three-stage mechanism for low-calcium aluminosilicate activation: (1) dissolution under strong alkali, releasing aluminate and silicate species; (2) polycondensation, initiating system gelation; and (3) reorganization into semi-crystalline zeolite-like phases for hardening [47]. Conversely, Davidovits [24] focused on NaOH/KOH-activated kaolin, proposing a “depolymerization-polycondensation” model where Si-O/Al-O bonds depolymerize in alkaline environments and then repolymerize into a -Si-O-Al- framework. Van Deventer et al. [25] expanded this to a four-stage process for kaolin and fly ash systems: (1) dissolution forming monomers; (2) monomer diffusion; (3) gel phase formation via polycondensation; and (4) dehydration-induced hardening. Fernández-Jiménez et al. [32] similarly identified four nonlinear stages for fly ash activation, noting early dissolution control followed by diffusion dominance. Zhuang et al. [17] simplified fly ash geopolymerization into alkali-promoted aluminate decomposition and polycondensation (Figure 3a). Duxson et al. [48] described a dynamic gel evolution: an initial aluminum-rich gel (Gel 1) transitions to a silicon-rich gel (Gel 2), culminating in a polysialate network, as depicted in the general conceptual model (Figure 3b). Similarly, Yao et al. [49] inferred a three-stage metakaolin geopolymerization—depolymerization, oligomerization, and polycondensation/hardening (Figure 3c) and Equations (1)–(4). These models highlight mechanistic diversity, reflecting material-specific reaction pathways.



$({si}_{2}O_{5}{,Al}_{2}O_{2})n+3nH_{2}O\overset{NaOH/KOH}{\to }n(OH{)}_{3}-Si-Al^{\left(-\right)}-(OH{)}_{3}$

(1)

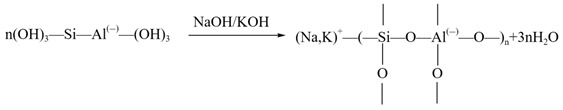

(2)

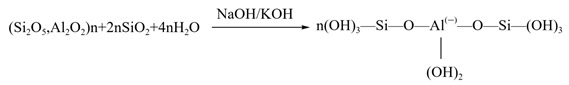

(3)

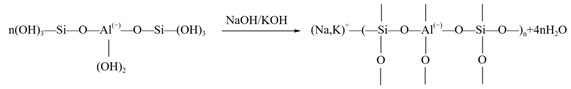

(4)

In summary, a consensus identifies the reaction process of AAP as comprising three stages: dissolution, depolymerization, and polycondensation. Specifically, raw materials dissolve under alkali activator action, depolymerize into monomeric units, and subsequently undergo polycondensation with hardening to form an inorganic gel network. This framework aligns with established models for low-calcium aluminosilicate systems and geopolymerization mechanisms. Collectively, while these mechanistic models differ in their specific pathways, a common distinction exists between high-calcium systems (e.g., slag), where C-A-S-H gel formation dominates, and low-calcium systems (e.g., fly ash, metakaolin), where N-A-S-H and zeolitic phases prevail. This compositional divergence fundamentally governs the differences in strength development kinetics, setting behavior, and long-term durability observed across AAP systems.

## 3. The Performance of Alkali-Activated Polymers

### 3.1. Compressive Strength

As a novel construction cementitious material, the mechanical properties of AAP have garnered significant interest, with compressive strength serving as a fundamental performance indicator. Studies have investigated compressive strengths across varied precursors, activators, curing temperatures, and durations. Barbosa et al. reported that metakaolin-based materials activated by alkaline solutions achieved 48.1 MPa after 1 h at 650 °C [26]. Sofi et al. observed 7-day and 28-day strengths in inorganic polymer concrete exceeding ordinary concrete by 10–15 MPa. Nazari et al. [50] attained 58.9 MPa by replacing 30% fly ash with rice husk ash under 80 °C curing for 28 days. Table 1 summarizes compressive strengths under diverse conditions, demonstrating rapid hardening, early strength, and high later-age performance. Key influencing factors include precursor alkali content, activator type and dosage, Si/Al ratio, and curing conditions. Komljenovic [51] compared activator efficacy via compressive strength at equivalent alkali dosage, identifying the hierarchy: KOH < NaOH + Na_2_CO_3_ < NaOH < NaOH + Na_2_SiO_3_. This was attributed to K^+^ ionic size limiting AlO_4_ bonding and Na_2_CO_3_’s insufficient alkalinity for precursor dissolution. Consequently, alkali-silicate combinations (e.g., sodium/potassium silicate) are widely adopted. However, commercial silicate activators—the costliest raw material component—significantly elevate production expenses, posing economic challenges for scalability.

The Si/Al ratio constitutes a critical compositional parameter governing strength development in AAP systems. An optimal Si/Al ratio in the range of 1.5–2.5 promotes the formation of a dense, well-cross-linked aluminosilicate network, maximizing compressive strength; ratios outside of this range result in either incomplete dissolution or excessive network rigidity, both of which are detrimental to mechanical performance.

### 3.2. Heat-Resistant Quality

**Figure 4:**
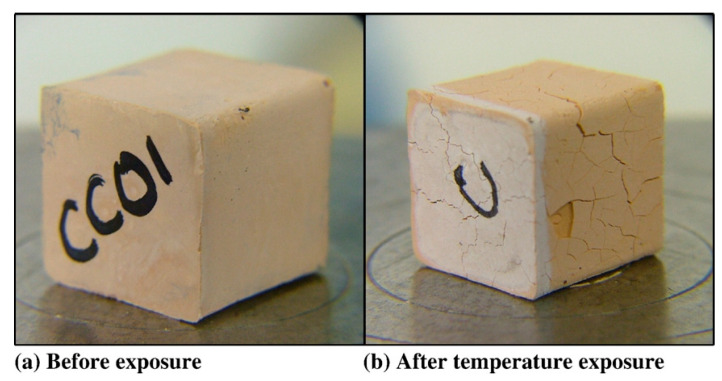
Photographs of metakaolin geopolymer specimens. (**a**) Before exposure, (**b**) after temperature exposure. Reprinted with permission from ref. [60].

**Figure 5:**
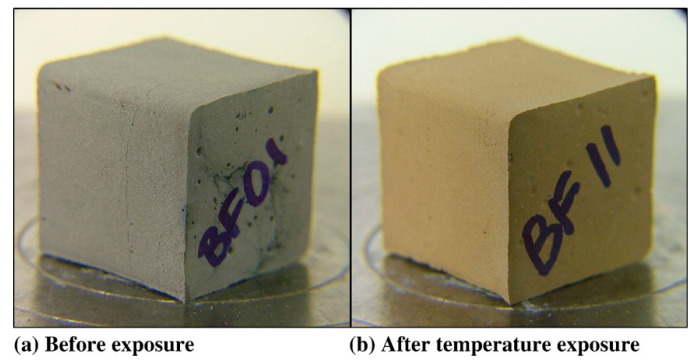
Photographs of fly ash geopolymer specimens. (**a**) Before exposure, (**b**) after temperature exposure. Reprinted with permission from ref. [60].

Alkali-activated polymers possess a stable three-dimensional network structure, exhibiting superior thermal stability. Van et al. [59] demonstrated that Na-PSS-type metakaolin-based specimens maintained structural integrity from 250 °C to 800 °C, with quartz/granite aggregates reducing thermal shrinkage to 1%. Rashad et al. [21] revealed that increased Na_2_O concentration densifies the matrix, enhancing compressive strength across temperatures; residual strength reached a minimum at 600 °C but recovered significantly at 1000 °C. Fly-ash-based alkali-activated polymers (FA-AAP) show exceptional thermal resistance, attributed to their low porosity and fine pore structure that impede water dissipation. Simultaneously, dispersed micropores provide moisture escape channels, mitigating structural damage. Kong et al. [60] compared FA-AAP and metakaolin systems; after 800 °C exposure, metakaolin specimens developed 0.1–0.2 mm (Figure 4) surface cracks (34% strength loss), whereas FA-AAP exhibited no cracking (Figure 5) and 6% strength gain. Pan et al. [61] further confirmed that higher fly ash content enhanced thermal resistance in fly ash/slag blends, with pure FA-AAP achieving peak strength at 600 °C; conversely, slag-rich specimens showed inferior performance, particularly at 300 °C. These studies collectively indicate that thermal resistance varies significantly with raw material composition and activator ratios. Owing to their exceptional stability, alkali-activated polymers present a viable alternative to conventional concrete for fire-resistant structures.

### 3.3. Anti-Freeze and Anti-Thawing Performance and Anti-Chemical Erosion Performance

Freeze–thaw durability and chemical erosion resistance are critical metrics for evaluating construction material longevity. Recent studies confirm that AAP exhibits significantly superior freeze–thaw resistance compared to Portland-cement-based systems. Freeze–thaw resistance tests in the reviewed studies were generally conducted following standard protocols, with temperature cycling between −18 °C and +5 °C and each cycle lasting approximately 2–4 h. Chemical erosion resistance was evaluated by immersing specimens in sulfate (Na_2_SO_4_) or acid solutions (H_2_SO_4_, HCl) at concentrations of 5–50 g·L^−1^ for durations ranging from 28 days to 365 days, with compressive strength retention and mass loss serving as the primary evaluation metrics. Sun et al. [62] demonstrated that after 300 freeze–thaw cycles, fly-ash-based AAP retained 95% compressive strength versus 80% for Portland cement, highlighting their structural resilience. This advantage is attributed to supplementary cementitious materials enhancing C-S-H, C-A-S-H, and N-A-S-H gel formation, which densify the microstructure and reduce water permeability. Fu et al. [28] subjected alkali-activated slag concrete to 300 cycles, observing minimal mass loss (0.12~0.70%) and ~90% retention of the relative dynamic modulus of elasticity without surface spalling. These metrics exceed the F300 grade standard (requiring >60% modulus retention after 300 cycles), confirming exceptional freeze–thaw resistance. Zhao et al. [63] further corroborated these findings while elucidating raw material composition effects on durability mechanisms.

Compared to Portland cement systems [64], alkali-activated polymers (AAP) exhibit superior durability in both acidic and alkaline environments, demonstrating exceptional stability under alkaline conditions. Palomo et al. [65] reported that alkali-activated metakaolin cement exhibited merely 1/13 and 1/12 the degradation rate of Portland cement in 5 wt% sulfuric acid and hydrochloric acid solutions, respectively. Owsiak et al. [66] further demonstrated that the waterglass-to-NaOH ratio critically regulates AAP durability in sulfate, hydrochloric acid, and acetic acid environments. This corrosion resistance originates from the absence of highly vulnerable phases (e.g., portlandite, calcium aluminate hydrates) in AAP reaction products. Alkali-activated slag and fly ash systems contain zeolitic phases alongside C-S-H gels and feldspathoids, while metakaolin-based systems exclusively form zeolite-like structures. Degradation mechanisms in highly acidic media involve depolymerization of aluminosilicate networks; release of silicate ions (SiO_4_^4−^); cation exchange (Na^+^/K^+^ replaced by H^+^); and dealumination of polymeric frameworks.

### 3.4. Anti-Penetrability Performance

**Figure 6:**
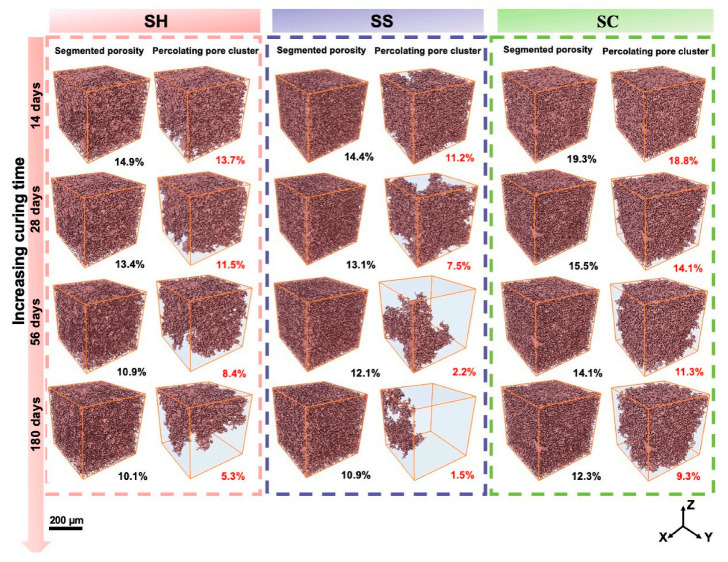
3D visualization and average segmented porosity (**left**) and percolating pore clusters (**right**) in each of the alkali-activated slag pastes with increasing curing time. Reprinted with permission from ref. [74].

Pores in hardened cement paste are classified by size as gel pores (1~10 nm), small capillary pores (10~50 nm), medium capillary pores (50~1000 nm), large capillary pores (1~10 μm), and macropores (>10 μm) [67]. The microporous structure, particularly large capillary pores and macropores, critically governs load-bearing capacity and material performance [68,69,70]. Pore architecture further dictates permeability and diffusivity, facilitating ingress of aggressive ions (e.g., ${CO}_{3}^{2-}$, ${SO}_{4}^{2-}$, ${Cl}^{-}$) that chemically degrade the binder matrix and accelerate corrosion, ultimately compromising durability [71,72]. Consequently, pore structure constitutes a key parameter controlling long-term performance. Alkali-activated cementitious materials exhibit lower porosity and a denser pore structure than Portland cement. Their pastes primarily contain pores <10 nm—dimensions that restrict fluid flow and ionic diffusion, significantly impeding external substance ingress. This structural characteristic confers exceptional impermeability. Shi [29] demonstrated that alkali-activated slag mortar outperforms Portland cement mortar in chloride resistance. The 28-day chloride ion permeability (electrical flux) of sodium-silicate-activated slag mortar was merely one-third that of Portland cement mortar, with Na_2_CO_3_ and NaOH activated systems showing even greater resistance. Hu et al. [73] systematically investigated activator composition effects on strength, shrinkage, and chloride migration resistance in alkali-activated slag mortars. Employing 4D X-ray micro-computed tomography (μCT) coupled with backscattered electron (BSE) imaging, X-ray diffraction (XRD), and thermogravimetric analysis (TGA), Su et al. [74] elucidated microstructure evolution in alkali-activated slag (AAS) pastes under three activators (sodium silicate, NaOH, Na_2_CO_3_) over 14~180 days. Their work established critical structure–property relationships between pore architecture and mechanical/impermeability performance (Figure 6 shows average porosity and percolated porosity). This provides a scientific foundation for designing low-carbon cementitious materials with tailored properties (e.g., high strength/durability) through activator selection from single-slag sources.

### 3.5. Setting Time

**Figure 7:**
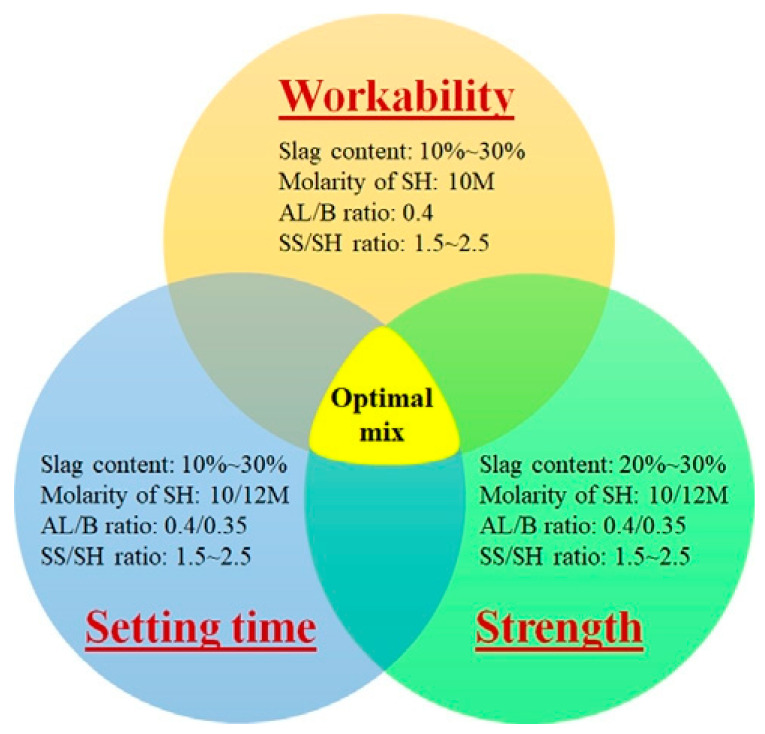
The optimal mixtures of AAFS concrete. Reprinted with permission from ref. [76].

Setting time represents a critical workability indicator for alkali-activated systems, which can be effectively tailored through activator parameters and raw material formulations. In alkali-activated slag (AAS) systems, Dodiomov’s study [46] demonstrated that adjusting the silica modulus (SiO_2_/Na_2_O ratio) and Na_2_O content enables broad control over setting behavior. Initial setting time ranged from 13 to 183 min and final setting time from 15 to 215 min while maintaining mortar fluidity between 133 and 230 mm, indicating excellent workability tunability. Sun et al. [75] systematically revealed synergistic effects of silica modulus, activator content, and the water-to-binder ratio on setting and strength development. Activator dosage and the water-to-binder ratio exhibited the most pronounced influence on setting kinetics. For fly ash–slag hybrid systems, Fang et al. [76] established that increased slag content and higher NaOH molar concentration significantly reduce setting times. Through comprehensive testing, they proposed an optimal formulation window: slag content: 20~30%; alkali liquid-to-binder ratio (AL/B): 0.4; NaOH concentration: 10 M; and sodium silicate-to-sodium hydroxide mass ratio (SS/SH): 1.5~2.5 (validated by performance data in Figure 7). These findings highlight the compositional synergy between raw materials. Ali et al. [77] corroborated that under ambient curing, increasing slag’s proportion in fly ash–slag blends enhances strength but accelerates setting, further evidencing the profound impact of precursor composition on hydration kinetics.

### 3.6. Contractility

Alkali-activated cementitious materials exhibit pronounced shrinkage. Alkali-activated slag cement (AASC) demonstrates significantly higher shrinkage than ordinary Portland cement (OPC) systems, with shrinkage-induced cracking representing one of the most critical barriers to wider adoption of this promising alternative binder. Neto et al. [78] revealed that autogenous shrinkage in alkali-activated slag binders substantially exceeds that of OPC. When activated by sodium silicate (modulus = 1.7) at 4.5% Na_2_O of slag mass, 21-day autogenous shrinkage was 4.5 times greater than Portland cement. This finding directs subsequent research toward mitigation strategies (e.g., shrinkage-reducing admixtures, mix optimization). Collins et al. [79] elucidated the fundamental mechanisms underlying the high drying shrinkage of AASC through comparative analysis with OPC concrete, explaining the anomalous moisture loss–shrinkage relationship. Their work established that microporous structure—particularly mesopore (2~50 nm) content and distribution—is more critical than macroscopic water loss in predicting shrinkage behavior, providing a theoretical foundation for pore structure engineering via activator design and chemical admixtures. Aydin et al. [45] demonstrated that a higher silica modulus correlates with increased drying shrinkage in AASC. When the modulus exceeds 0.4, shrinkage escalates with rising Na_2_O content. Notably, at modulus = 1.2 and 6% Na_2_O, AASC reached 4.0 × 10^−3^ drying strain at 3 days—eightfold higher than OPC’s < 0.5 × 10^−3^. Ye et al. [80] further established through controlled-humidity experiments that activators govern shrinkage via pore structure modulation, while the highly viscoelastic solid skeleton contributes to intense shrinkage susceptibility. Collectively, these studies confirm that activator type and dosage are pivotal factors controlling shrinkage, directly impacting material design and durability assessment.

Raw material properties (type, particle size), activator characteristics (type, modulus, alkali equivalent), water content, and curing conditions collectively govern the strength, workability, and setting behavior of alkali-activated polymers. These materials offer compelling advantages: rapid hardening and early-age strength development; high ultimate mechanical strength; superior thermal stability; excellent freeze–thaw resistance; enhanced acid corrosion resistance; and low permeability. However, excessive shrinkage remains a persistent challenge constraining broader implementation. These performance characteristics collectively address the core technical requirements of grouting engineering outlined in the Introduction, namely, rapid gelation, early strength gain, durability under aggressive environments, and low permeability.

### 3.7. Effect of Raw Material Types on AAP Properties

The properties of alkali-activated polymers are strongly governed by the type of precursor material employed. Table 2 summarizes the primary performance characteristics associated with the main raw material categories used in AAP systems.

In general, high-calcium precursors such as slag promote rapid strength development through C-A-S-H gel formation but are accompanied by significant shrinkage. Low-calcium precursors such as fly ash and metakaolin form N-A-S-H and zeolitic phases, conferring superior thermal stability and chemical resistance with lower shrinkage. Binary or ternary precursor combinations are therefore widely adopted in practice to balance these competing performance characteristics and meet the specific demands of grouting engineering applications.

## 4. Application in the Grouting Process

### 4.1. Grouting Reinforcement Application

Soft soil stabilization is essential in geotechnical engineering, where conventional cement-based grouts exhibit limited durability under sulfate/chloride attack and high carbon footprint [81]. Alkali-activated geopolymers derived from industrial byproducts offer a sustainable alternative, gaining significant attention for their dual economic and environmental benefits [11,82,83]. Moreover, previous studies [11,82,83] systematically reviewed waste-derived alkali-activated grouts to establish foundations for practical implementation, highlighting their efficacy in mitigating soil contamination risks while conserving land resources.

**Figure 8:**
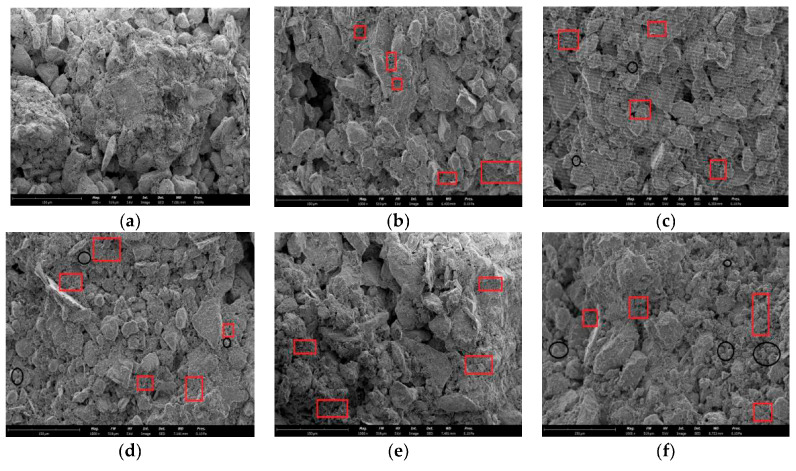
SEM image of solidified soil with different polymer contents after curing period of 28 d. The content of geopolymer: (**a**) 0% (**b**) 5% (**c**) 10% (**d**) 15% (**e**) 20% (**f**) 25%. The red boxes and black circles indicate the formed gels and pores/unreacted particles, respectively. Reprinted with permission from ref. [86].

Alkali-activated polymers demonstrate significant potential as low-carbon cementitious materials for soft soil stabilization, with optimized precursor-activator formulations enhancing geotechnical performance. Zhang et al. [84] demonstrated that metakaolin-based systems induce ductile failure behavior and low shrinkage deformation in soft clay while significantly improving compressive strength (up to 3.2 MPa), elastic modulus (≥120 MPa), and failure strain in sulfate-rich environments. Notably, these systems reduced expansion strain by >50% compared to lime stabilization, effectively mitigating salt heave risks. For fly-ash-based systems, Rios et al. achieved unconfined compressive strength (UCS) of 5.2 MPa at 15% dosage in silty sand [33], while Noor et al. documented UCS increases from 0.3 MPa to 1–8 MPa with exceptional acid/chloride resistance [34]. Liu et al. [85] further established that KOH activators outperformed NaOH in fly-ash-stabilized loess, attaining 28-day UCS of 7.0 MPa through gel-induced microstructural densification. SEM and XRD analysis revealed that the hydration of steel slag–fly ash geopolymer produces C-S-H and C-A-S-H cementing materials, which effectively fill the gaps between soil particles and enhance the mechanical properties of solidified soil [86]. Figure 8 presents a microstructure diagram of solidified soil mixed with different geopolymers after 28 days of curing [86]. SEM observations confirm that the macroscopic properties of steel slag–fly ash geopolymer primarily originate from the steel slag–fly ash substance in the geopolymer. Under the alkali excitation of water glass, a depolymerization–condensation reaction occurs, forming an amorphous phase C-S-H and C-A-S-H flocculent gel that combines with the vitreous body in the geopolymer. This flocculent gel material strengthens the binding force, enhancing the compactness and compressive strength of the solidified soil.

In hybrid waste systems, Mariana et al. [87] reported that sugarcane bagasse–eggshell ash composites achieved 5 MPa compressive strength at 30% moisture content, with elevated curing temperature (40 °C) and extended duration (28 days), enhancing microstructural density. Wang et al. [30] developed slag–fly ash–waste soil grouts exhibiting tunable rheology and strength through activator optimization, enabling precise control over injectability. Furthermore, Liu et al. [88] utilized industrial byproducts—fly ash (FA), ground granulated blast furnace slag (GGBS), and silica fume (SF)—to fully replace cement, preparing a novel alkali-activated backfill grout. Their research demonstrated that higher GGBS/SF contents, elevated alkali dosage, or reduced alkali activator solution modulus (MAAS) and liquid-to-solid ratio (LSR) facilitated timely setting and hardening for ground settlement mitigation, albeit at the expense of flowability compromise.

Recent studies have further advanced the practical application of AAP grouting materials. Wang et al. demonstrated that GGBFS–zeolite powder grouting materials activated by NaOH achieved excellent early strength and setting control for silty mudstone slope reinforcement [89]. He et al. further validated geopolymer grouting materials incorporating fly ash, slag, and steel slag in pile foundation post-grouting, confirming their superior mechanical performance and environmental durability under field conditions [90].

Owing to their exceptional comprehensive performance—including rapid setting, early strength development, high mechanical properties, thermal stability, freeze–thaw resistance, acid corrosion resistance, and low permeability—alkali-activated polymers are recognized as low-energy, low-carbon, green cementitious alternatives. These materials demonstrate significant potential for solid waste solidification/stabilization and grouting engineering applications, particularly in resource-efficient utilization of industrial byproducts. Nevertheless, research on their use in soil stabilization remains preliminary due to the inherent complexity and heterogeneity of soil composition, structure, and engineering properties. Current studies predominantly focus on the mechanical properties (e.g., unconfined compressive strength, deformation modulus) of polymer-stabilized soils. Crucially, systematic investigations into durability evolution mechanisms under conventional and complex environmental conditions—such as wet–dry cycles, freeze–thaw cycles, chemical erosion, and long-term permeation—are notably deficient. Consequently, future research must prioritize long-term durability performance to enable practical implementation.

### 4.2. Grouting Water-Blocking Application

AAP demonstrates significant advantages as a low-carbon grouting material for seepage control and corrosion protection engineering. However, its long-term durability under harsh environments involving flowing groundwater and chemical erosion presents critical challenges. Groundwater ingress can trigger depolymerization of the aluminosilicate network (“water corrosion”) in hardened grouts, leading to mechanical degradation [91]. As noted by Bentz and Garboczi [92], calcium hydroxide in Portland cement systems is susceptible to leaching under hydrodynamic conditions, compromising soil stabilization strength. In contrast, AAP exhibits superior aqueous corrosion resistance due to the absence of soluble calcium hydroxide phases. Nevertheless, their aluminosilicate frameworks remain vulnerable to ionic leaching in high-flow scenarios. Impermeability serves as a vital performance metric for AAP grouts, quantifying resistance to fluid/ion penetration through stabilized matrices under hydraulic, chemical, or electrochemical gradients. High-impermeability grouted soils feature densified microstructures that effectively impede external fluid ingress and corrosive ion diffusion. Consequently, impermeability and volumetric stability constitute dual critical criteria for evaluating AAP grouting efficacy in subsurface environments.

Extensive research has focused on the impermeability and volumetric stability of AAP. Borçato et al. [93] established that increasing sodium methoxide (CH_3_ONa) content in AAP grouts nonlinearly reduces shrinkage rates, with diminishing effects beyond 30% concentration. Li et al. [94] investigated the water-to-cement ratio and sodium silicate-to-waterglass ratio (Solution A/B) in cement–sodium silicate binary grouts, achieving a maximum impermeability pressure of 1.6 MPa under standardized hydraulic gradients. Furthermore, Song et al. [95] demonstrated that optimizing activator concentration and liquid-to-solid ratio enables AAP formulations to attain exceptional water resistance; laboratory-tested specimens exhibited a >0.80 submerged-to-air strength ratio and >0.8 MPa impermeability pressure after 28-day curing. These findings underscore the critical role of compositional parameters in enhancing AAP durability for seepage control applications.

Sodium silicate (Na_2_SiO_3_) serves as a critical alkaline activator in alkali-activated materials (AAM), significantly reducing setting time and enhancing waterproofing capabilities—particularly vital for grouting in water-rich strata. Beyond accelerating cement hydration and improving grout mechanical properties [37,38], sodium-silicate-modified grouts exhibit rapid initial setting, high strength, and resistance to dilution under groundwater exposure. Nevertheless, sodium silicate incorporation may induce mortar shrinkage due to reduced [SiO_4_] dissolution and weakened gel networks [96,97,98], compromising long-term durability. To mitigate shrinkage, chemical admixtures or supplementary cementitious materials are integrated via binary grouting systems, enhancing erosion resistance [99]. For instance, Yan et al. [39] addressed gelation instability and poor groundwater resistance in Portland cement–waterglass systems by incorporating methylcellulose, establishing an optimized anti-washout model. Similarly, Xu et al. [31] replaced cement with steel slag and ground granulated slag, employing retarders to control gelation kinetics. Despite these advancements, binary grouting still faces challenges, including pipeline clogging, overgrouting, and incomplete void filling.

The performance of grouting materials under dynamic water conditions critically governs the long-term efficacy of water-sealing projects. Lin et al. [100] enhanced composite grout performance by optimizing cement–sodium silicate blends with sulphoaluminate cement and slag, effectively mitigating scouring effects in flowing water. Furthermore, Wu et al. [101] employed a response surface methodology to develop an eco-efficient binary grout using fly ash cement and sodium silicate, demonstrating optimal efficacy for sealing fissure water influx in tunnels. Song et al. [102] pioneered a two-component industrial waste-based grout with tunable gelation time (seconds to minutes) and exceptional water corrosion resistance. Collectively, alkali-activated polymer grouts—characterized by rapid gelation, superior anti-washout resistance, and high impermeability pressure—demonstrate good suitability for leakage control in water-rich strata.

## 5. Conclusions

As a low-energy, low-carbon-emission, green cementitious material, alkali-activated polymers exhibit significant application potential in solid waste utilization and grouting engineering. This review synthesizes their reaction mechanisms, performance characteristics, and grouting applications (soil stabilization and water sealing), yielding the following key conclusions:

(1) The alkali activation process comprises three sequential stages: dissolution of raw materials under alkaline conditions, depolymerization into monomeric species, and polycondensation forming hardened inorganic gel matrices.
(2) Material properties (precursor type, particle size) and process parameters (activator type, silica modulus, alkali equivalent, water demand, curing conditions) collectively govern strength development, workability, and setting behavior.
(3) By leveraging superior properties—including rapid setting, early strength gain, high-temperature stability, freeze–thaw resistance, acid/salt corrosion resistance, and low permeability—these materials are extensively applied in ground stabilization and hydraulic sealing projects while reducing CO_2_ emissions by 60–80% compared to ordinary Portland cement, offering a viable low-carbon pathway for large-scale infrastructure development. Collectively, AAPs represent a sustainable alternative for infrastructure resilience in challenging environments.

## 6. Future Perspective

Extensive fundamental research has been conducted globally on alkali-activated polymers, yet critical knowledge gaps persist in optimizing formulations and applications:

(1) Performance variability stemming from divergent raw material sources necessitates source-specific formulations. Significant fluctuations in mechanical properties and workability hinder reliable implementation, particularly for geographically diverse precursors.
(2) Despite exceptional properties—including rapid hardening, early strength, corrosion resistance, impermeability, and thermal stability—pronounced shrinkage induces microcracking risks, critically constraining advancement.
(3) Systematic understanding of long-term durability mechanisms under complex environmental stressors (wet–dry cycles, freeze–thaw exposure, chemical erosion, prolonged permeation) remains limited. Future work must prioritize accelerated aging studies and microstructural evolution modeling.
(4) While research on geopolymer cementitious materials progresses rapidly, achieving systematic engineering implementation requires synergistic collaboration across civil engineering, materials science, and environmental engineering disciplines to bridge theoretical advances with field applications.

## Figures and Tables

**Table 1:** The compressive strength of typical alkali-initiated polymer materials.

Reference	Precursor Materials	Alkali Activator	Aggregate/Admixture	Maintenance Temperature/°C	Compressive Strength/MPa
[52]	Weathered Granite + Fly Ash	Na_2_SiO_3_ + NaOH		60	4.62~18.42 (7 d)
[53]	Metakaolin	Na_2_SiO_3_		25~30	3.58~78.96 (7 d)
[54]	Metakaolin + Zeolite	Na_2_SiO_3_ + NaOH	Softwood waste residue	50 (24 h)+ room temperature	7.41 + 15.84 (28 d)
[55]	Industrial Sludge	Na_2_SiO_3_ + NaOH		room temperature	34.70~49.40 (7 d)
[56]	Red Mud + Fly Ash	Na_2_SiO_3_ + NaOH		room temperature	18.20~43.10 (28 d)
[57]	Rice Husk Ash	NaOH	Sand	80 (24 h)+ room temperature	28.56~39.95 (28 d)
[58]	Metakaolin + High-Calcium Fly Ash	Na_2_SiO_3_ + NaOH	Recycled coarse aggregate, river sand	60 (2 d) + 22~25	32.90~47.20 (7 d)

**Table 2:** Effect of raw material types on the key properties of AAP.

Raw Material	Primary Performance Advantage	Typical Effect
Slag	Compressive strength, setting time	Rapid early strength gain; however, pronounced autogenous and drying shrinkage
Fly ash	Thermal resistance, impermeability	Superior performance at elevated temperatures (up to 800 °C); low shrinkage
Metakaolin	Compressive strength, chemical resistance	High mechanical strength; excellent acid and sulfate corrosion resistance
Slag + fly ash	Balanced comprehensive performance	Tunable setting time and strength through slag/fly ash ratio adjustment
Slag + metakaolin	Shrinkage mitigation	Metakaolin addition effectively reduces autogenous shrinkage of slag-based systems

## Data Availability

The original contributions presented in this study are included in the article. Further inquiries can be directed to the corresponding authors.

## Abbreviations

The following abbreviations are used in this manuscript:

AAP	alkali-activated polymers
FA-AAP	Fly-ash-based alkali-activated polymers
XRD	X-ray diffraction
SEM	Scanning Electron Microscope
BSE	backscattered electron
TGA	thermogravimetric analysis
AAS	alkali-activated slag
SS/SH	sodium silicate-to-sodium hydroxide mass ratio
AL/B	alkali liquid-to-binder ratio
AASC	alkali-activated slag cement
OPC	ordinary Portland cement
FA	fly ash
GGBS	ground granulated blast furnace slag
SF	silica fume
LSR	liquid-to-solid ratio
MAAS	alkali activator solution modulus
AAM	alkali-activated materials
